# Bioinformatics Combined With Biological Experiments to Explore the Promotion of Lung Metastasis by CCL18 in the Immune Microenvironment of Colorectal Cancer

**DOI:** 10.1155/humu/1943814

**Published:** 2026-05-20

**Authors:** Qingcheng Xia, Shuting Yin, Jiping Chen, Haonan Wang, Yexin Shi, Jie Li, Zidong Yuan, Zijun Wang, Ming Lu, Qinghong Zhao, Wei Wei, Xiang Ma

**Affiliations:** ^1^ Department of General Surgery, The Second Affiliated Hospital of Nanjing Medical University, Nanjing, China, njmu.edu.cn; ^2^ Department of General Surgery, The Affiliated Jiangsu Shengze Hospital of Nanjing Medical University, Suzhou, China, njmu.edu.cn; ^3^ Department of General Surgery, Affiliated Hospital of Nantong University, Nantong, China, ahnmc.com; ^4^ Department of General Surgery, Sir Run Run Hospital, Nanjing Medical University, Nanjing, China, njmu.edu.cn; ^5^ Department of Emergency and Pediatric Intensive Care Unit (PICU), Children′s Hospital of Nanjing Medical University, Nanjing, China; ^6^ Department of Oncology, The Second Affiliated Hospital of Nanjing Medical University, Nanjing, China, njmu.edu.cn

## Abstract

**Background:**

Colorectal cancer (CRC) is one of the most prevalent malignant neoplasms. Tumor metastasis represents a significant contributor to unfavorable prognosis, and lung metastasis is the most common extra‐abdominal metastasis of CRC. However, pulmonary metastatic CRC has not received significant attention. Therefore, in this study, our main aim was to discover the key genes for lung metastasis and improve the prognosis of CRC.

**Methods:**

Differentially expressed genes (DEGs) between primary tumors and patients with pulmonary metastatic CRC were obtained by analysis of a comprehensive database of gene expression (GEO). DEGs were screened by prognostic survival analysis, TCGA differential expression analysis, and single gene GSEA.

**Results:**

Combining the differences in patient prognosis and expression, we found that CCL18 is a key gene in CRC lung metastasis. Macrophage coculture and transwell assay verified the potential of CCL18 in promoting the invasive metastatic ability of CRC cells, as well as the proliferation of CRC cells.

## 1. Introduction

Colorectal cancer (CRC) is a prevalent malignant tumor with high mortality globally [1, 2]. Although surgical resection and chemotherapy can cure early‐stage CRC, most patients present with advanced disease and distant metastases, resulting in poor outcomes [3, 4]. Tumor metastasis, particularly to the liver and lungs, is the leading cause of CRC‐related death [5]. Lung metastasis occurs in 20%–30% of metastatic CRC cases and often portends a worse prognosis than liver metastasis [6]. However, the biology of CRC lung metastasis is less understood and has garnered insufficient attention [7, 8]. Early detection and targeted intervention for lung metastatic CRC are urgently needed to improve patient survival [9].

CRC tumors do not exist in isolation but are embedded in a complex tumor microenvironment (TME) composed of stromal cells, blood vessels, and infiltrating immune cells [10, 11]. The TME plays a critical role in cancer progression by fostering interactions between cancer cells and immune components [12–14]. Tumor‐associated macrophages (TAMs) are among the most abundant immune cells in the TME and are pivotal regulators of tumor behavior. TAMs originate from circulating monocytes that infiltrate tumors and differentiate in situ under tumor‐derived signals. Importantly, TAMs display remarkable plasticity, generally polarizing toward either a proinflammatory “M1” phenotype or an anti‐inflammatory “M2” phenotype, though intermediate states also exist [15]. Classically activated M1 macrophages secrete inflammatory cytokines and mediate antitumor immunity. In contrast, alternatively activated M2 macrophages produce immunosuppressive factors and promote tissue remodeling, angiogenesis, and tumor growth. In solid tumors, TAMs are frequently skewed to an M2‐like state, contributing to tumor immune evasion and metastasis. Indeed, M2‐polarized TAMs stimulate angiogenesis, enhance metastasis, and repress antitumor immune responses, underscoring their protumoral role in the TME. By contrast, M1 macrophages in tumors tend to exert tumoricidal effects; their presence is often associated with improved immune surveillance, although their numbers are usually lower in advanced tumors due to the immunosuppressive milieu. This functional dichotomy of macrophages sets the stage for understanding how specific TAM‐derived factors influence metastasis.

Chemokines produced in the TME orchestrate immune cell recruitment and also act directly on cancer cells [16]. Among TAM‐secreted mediators, the CC chemokine ligand 18 (CCL18) stands out as an M2 macrophage‐associated chemokine implicated in cancer progression. CCL18 is primarily expressed by human macrophages and immature dendritic cells. In vitro, macrophages polarized with IL‐4/IL‐13 (M2‐like) secrete high levels of CCL18, whereas classically activated M1 macrophages produce little or no CCL18 under normal conditions [17]. Notably, extreme inflammatory stimulation can induce atypical CCL18 expression in M1 macrophages, but this is considered a nonphysiologic artifact of intense LPS exposure. In actual tumor settings, M2‐like TAMs are the predominant source of CCL18, consistent with their immunosuppressive, protumor phenotype. M1‐polarized macrophages might express CCL18 at low levels, but within tumors, they mainly exert antitumor functions and are not responsible for the elevated CCL18 levels observed in advanced cancers. For these reasons, our study focuses on CCL18 derived from M2 TAMs as a key factor in CRC lung metastasis. We hypothesize that TAM‐secreted CCL18 in the lung metastatic microenvironment drives tumor invasiveness and immune escape, whereas tumor cells themselves contribute minimally to CCL18 production.

Growing evidence indicates that CCL18 is a crucial mediator of tumor progression across multiple cancer types. This primate‐specific chemokine can reprogram the immune microenvironment by recruiting regulatory T cells (Tregs) and reinforcing macrophage polarization toward the protumoral M2 state. CCL18 is known to directly enhance cancer cell invasion, epithelial–mesenchymal transition (EMT), and angiogenesis, thereby promoting metastasis. For example, breast cancer TAMs abundantly produce CCL18, which binds to its receptor PITPNM3 on cancer cells, triggering integrin clustering and facilitating extravasation and metastasis [18]. High CCL18 levels in breast tumor stroma or blood correlate with lymph node metastasis and reduced patient survival. Similarly, in head and neck cancers, elevated serum CCL18 is associated with advanced tumor stage, lymph node metastasis, and shorter overall survival (OS). CCL18′s immunosuppressive functions, such as induction of Tregs, further enable tumors to evade immune surveillance. Notably, CCL18 expression is highest at the invasive front of tumors, and high circulating CCL18 often denotes aggressive disease with poor prognosis. These insights suggest CCL18 is a pivotal prometastatic factor and a potential therapeutic target in the TME.

## 2. Methods

### 2.1. Data Sources and Sample Characteristics

We obtained three independent gene expression microarray datasets (Series GSE41258, GSE68468, and GSE131418) from the NCBI Gene Expression Omnibus (GEO) database for bioinformatics analyses. Each dataset contains primary CRC tumor samples and metastatic CRC samples, enabling direct comparisons relevant to lung metastasis:

• GSE41258: This dataset (Affymetrix Human Genome U133A platform, GPL96) includes *n* = 390 expression profiles from CRC patients. The samples encompass primary colon adenocarcinomas, adenomatous polyps, normal colon mucosa, and distant metastases (liver and lung). From GSE41258, we specifically analyzed 186 primary CRC tumor samples versus 20 CRC lung metastasis samples (resected pulmonary metastatic lesions) to identify lung metastasis–associated genes. All raw data were background corrected and normalized as per GEO′s standard pipeline.
• GSE68468: This dataset (Affymetrix U133A, GPL96) provides *n* = 386 CRC patient samples. It comprises 189 primary colon tumor specimens and 20 lung metastatic CRC specimens, along with other sample types (normals, polyps, etc.). We extracted the primary tumor vs lung metastasis subset (189 vs. 20 samples) for differential expression analysis, analogous to GSE41258. Notably, the lung metastasis samples in GSE68468 were obtained from surgical resections of CRC pulmonary metastatic nodules, matched with primary tumors from the same patients in many cases.
• GSE131418: This is a large compendium (Rosetta/Merck Human RSTA Custom 2.0 microarray, GPL15048) combining two cohorts of CRC patients. In total, it contains 878 primary CRC tumors and 257 metastatic CRC lesions. The dataset was designed to profile transcriptomic differences between primary tumors and distant metastases while accounting for tissue‐specific effects. We leveraged GSE131418 to validate gene expression trends in metastases on a larger scale. Specifically, we focused on samples annotated as lung metastases within this dataset. By integrating this large dataset, we sought to identify robust differentially expressed genes (DEGs) associated with CRC lung metastasis.

### 2.2. Differential Gene Expression and Identification of CCL18

We performed transcriptome differential expression analysis between primary tumor and lung metastasis groups using the R limma package. For each dataset, probes with |log2(*f* *o* *l* *d* *c* *h* *a* *n* *g* *e*)| > 1 and *p* < 0.05 were considered significantly DEGs. We visualized DEGs using heat maps and volcano plots generated in R (ggplot2 and ggpubr) to ensure that lung metastasis samples clustered distinctly from primaries based on the top DEGs. Next, we cross‐compared the DEGs from all three GEO datasets to find consistently dysregulated genes in CRC lung metastases. Venn diagram analysis identified overlapping upregulated and downregulated DEGs common to GSE41258, GSE68468, and GSE131418 (Table 1).

To further narrow the candidates, we evaluated their prognostic relevance. Kaplan–Meier survival analyses were conducted using the KMplot online tool to test whether each candidate gene′s expression correlated with OS or recurrence‐free survival (RFS) [19]. Genes whose high expression significantly associated with worse survival in CRC (log‐rank *p* < 0.05) were prioritized as potential metastasis‐promoting genes. In parallel, we validated differential expression of candidates using The Cancer Genome Atlas (TCGA) CRC cohort. We utilized the GEPIA web server to compare candidate gene expression in TCGA‐COAD/READ (colon and rectal adenocarcinoma) versus normal tissues (GTEx), ensuring the direction of dysregulation (up or down in tumor) matched our observations [20].

### 2.3. Gene Set Enrichment and Immune Infiltration Analysis

To explore CCL18′s functional context, we performed single‐gene gene ontology and pathway enrichment analyses on TCGA CRC data. Using the LinkedOmics platform, we conducted gene set enrichment analysis (GSEA) correlating CCL18 expression with Hallmark pathways and GO biological processes. Enrichment with false discovery rate (FDR) < 0.05 was considered significant [21]. We also assessed immune cell infiltration levels in CRC tumors relative to CCL18 expression. We downloaded precomputed immune infiltration scores for TCGA‐COAD/READ from SangerBox (an online platform integrating ESTIMATE and ssGSEA methods). Specifically, we extracted macrophage infiltration scores and M1/M2 subtype signatures [22–24]. Pearson′s correlation coefficients between CCL18 expression and various immune cell abundance scores were calculated. This analysis allowed us to identify which immune cell populations track with CCL18 in CRC tissues.

### 2.4. Cancer Cell Line Expression Analysis

One critical question raised during our analysis was whether CCL18 could be expressed by CRC epithelial cells themselves, or whether it is exclusively from infiltrating immune cells. To address this, we interrogated the Cancer Cell Line Encyclopedia (CCLE) database. CCLE provides transcriptomic data for hundreds of human cancer cell lines, including dozens of CRC cell lines.

### 2.5. Cell Culture

THP‐1, HCT116, and SW480 cells were obtained from the Cell Bank of the Chinese Academy of Science and maintained in complete medium containing 10% fetal bovine serum (Gibco) and 1% penicillin–streptomycin (invitrogen); THP‐1 and HCT116 were cultured using RPMI 1640 (Gibco), and SW480 was cultured using DMEM (Gibco). Well‐cultured THP‐1 were differentiated into macrophages by incubation with 150‐nM phorbol 12‐myristate 13‐acetate (PMA, Selleck, China, S7791) for 24 h. Macrophage M2 polarization was obtained by incubation with 20 ng/mL IL‐4 (MCE, China, HY‐P70445) and 20 ng/mL IL‐13 (MCE, China, HY‐P70568).

### 2.6. siRNA transfection

Cells were spread in six‐well plates 24 h in advance so that the cell density was around 60%–70% before transfection. Use jetPrime (Polyplus) transfection reagent and perform siRNA transfection according to the instructions.

### 2.7. ELISA

Extract the supernatant of cell culture fluid, centrifuge at 1000 rcf for 20 min, take the supernatant after centrifugation, and perform Elisa test according to the instruction of the Elisa kit.

### 2.8. Real‐Time Quantitative Fluorescent PCR

All cell samples are fresh, not fixed, and under macrodissection. Each group had three samples at least. Total RNA was extracted using Trizol (invitrogen). The concentration and quality of RNA were detected using a One Drop OD‐1000 spectrophotometer, with an A260/280 ratio within the range of 1.8–2.0. Subsequently, 1000 ng of RNA was converted into cDNA in a 20‐*μ*L reaction mixture by using Hiscript III Reverse Transcriptase (Vazyme, Nanjing, China). The reaction procedure of reverse transcription was set to 50°C for 15 min and 85°C for 5 s. RT‐qPCR was performed using a LightCycler 480 Real‐Time PCR Detection System (Roche, Switzerland) and Taq Universal SYBR qPCR Master Mix (Vazyme), following the manufacturer′s protocol. There was no Cq value for the amplification of the no template control (NTC). Gene‐specific primers, listed in Table 2, were designed with amplicon lengths of around 100 bp and confirmed using NCBI blasting. The relative expression of target genes was calculated using the 2 − △△CT method, with beta‐actin serving as the internal reference. Each qPCR assay was retained with an efficiency of 90%–110%, *R* ^2^ ≥ 0.99. We identified LOD = 1.5 targeting molecules with a 95% confidence interval by testing 10‐fold serial dilutions of characterized stocks of in vitro transcribed RNA. Each sample underwent two or three technical replicates to ensure robust results.

### 2.9. Transwell Cell Invasion Assay

The cells were digested and counted, and at the standard of 5000–10,000 cells per well, the cells were evenly spread in the cell chambers that were coated with matrix gel in advance, the chambers were serum‐free medium, and complete medium was added to the culture plate in which the chambers rested. After 24 h, the chambers were removed, the cells were fixed using 4% paraformaldehyde, stained using crystal violet staining solution, and photographed for counting.

### 2.10. Cell Proliferation and Viability Assays

CRC cells were cultured either in conditioned medium from M2 macrophages (with high CCL18) or in control medium, and growth was measured by CCK8 assay and cell counting over 96 h.

### 2.11. Statistical Analysis

Statistical analysis was performed with GraphPad Prism (Version 8.0) and SPSS software (v 23.0). Student′s *t*‐test or *χ*2 test was used to assess statistical significance between groups. All experimental results were presented as mean SD, and we have independently repeated all experiments at least three times. *p* < 0.05 was considered significant.

## 3. Results

### 3.1. DEGs in CRC Lung Metastases

Using the GEO discovery datasets, we identified a set of genes dysregulated in CRC lung metastases compared with primary tumors. Figure 1A–C shows heat maps and volcano plots for DEGs in GSE41258, GSE68468, and GSE131418, respectively. Each dataset yielded dozens of significant DEGs (adjusted *p* < 0.05, |logFC| > 1). When we overlapped the DEG lists, we found five upregulated and three downregulated genes common to all three datasets (Figure 1D–E and Table 1). These overlapping DEGs likely represent a lung metastasis‐specific signature in CRC.

Figure 1Screening of differentially expressed genes between colorectal cancer primary tumors and lung metastases. (A) Heat map and volcano plot of GSE41258 using R. (B) Heat map and volcano plot of GSE68468 using R. (C) Heat map and volcano plot of GSE131418 using R. (D) Overlapping upregulated DEGs in GSE41258, GSE68468, and GSE131418. (E) Overlapping downregulated DEGs in GSE41258, GSE68468, and GSE131418.

**Figure:**
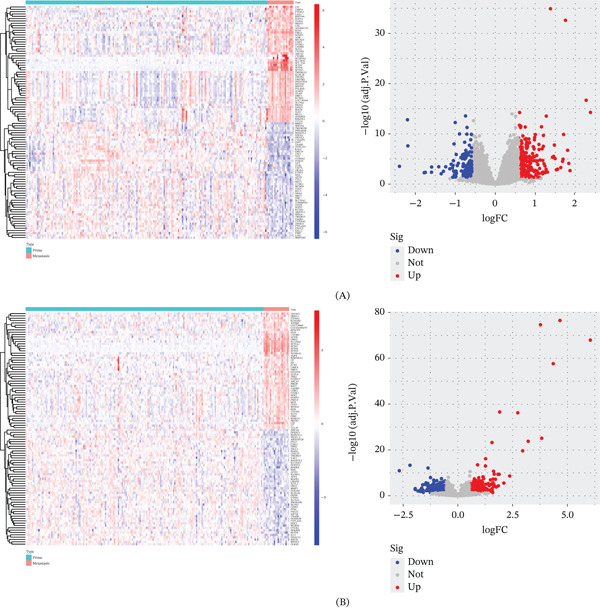
(A)

**Figure:**
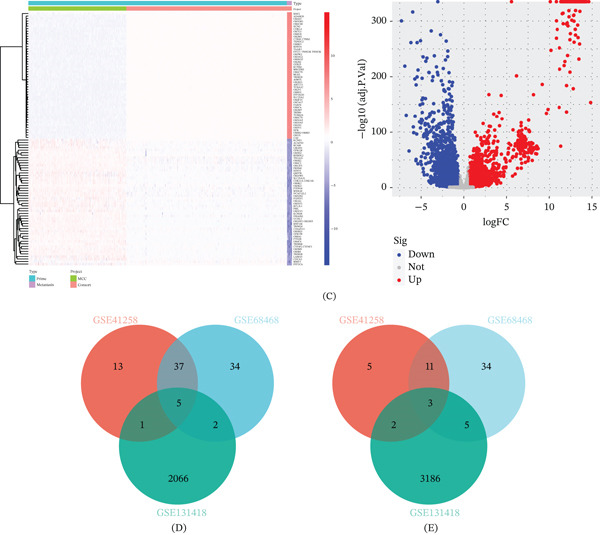
(B)

**Table 1:** Significantly overlapping of upregulated and downregulated DEGs.

Gene symbol	NCBI ID	Gene name	Up/down
C3	718	Complement C3	Up
CCL18	6362	C‐C motif chemokine ligand 18	Up
FMO2	2327	Flavin containing dimethylaniline monoxygenase 2	Up
SFTPA2	729238	Surfactant protein A2	Up
SFTPD	6441	Surfactant PROTEIN D	Up
ACTG2	72	Actin gamma 2, smooth muscle	Down
EXOC5	10640	Exocyst complex component 5	Down
MMP3	4314	Matrix metallopeptidase 3	Down

**Table 2:** Primers for RT‐qPCR.

ID	Sequence (5 ^′^–3 ^′^)
*β*‐actin F	ATCGTGCGTGACATTAAGGAGAAC
*β*‐actin R	AGGAAGGAAGGCTGGAAGAGTG
CCL18 F	GTTGACTATTCTGAAACCAGCCC
CCL18 R	GTCGCTGATGTATTTCTGGACCC
CCL22 F	TCCTGGGTTCAAGCGATTCTCC
CCL22 R	GTCAGGAGTTCAAGACCAGCCT
TGF*β* F	TACCTGAACCCGTGTTGCTCTC
TGF*β* R	GTTGCTGAGGTATCGCCAGGAA
IL‐10 F	TCTCCGAGATGCCTTCAGCAGA
IL‐10 R	TCAGACAAGGCTTGGCAACCCA

### 3.2. Association of Pivotal Genes With CRC Prognosis

**Figure 2:**
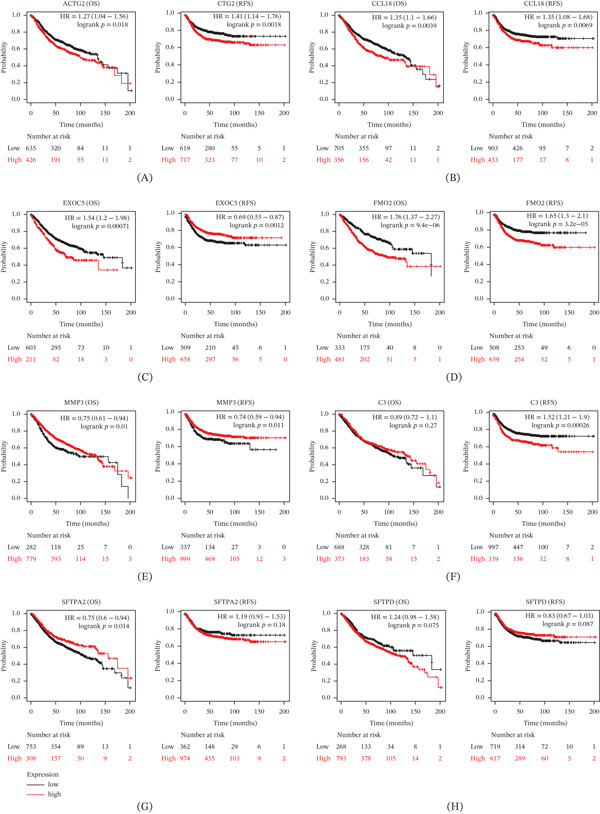
Association of pivotal genes with colorectal cancer prognosis. (A) OS (overall survival) and RFS (recurrence‐free survival) in CRC patients with different expression of ACTG2. (B) OS and RFS in CRC patients with different expression of CCL18. (C) OS and RFS in CRC patients with different expression of EXOC5. (D) OS and RFS in CRC patients with different expression of FMO2. (E) OS and RFS in CRC patients with different expression of MMP3. (F) OS and RFS in CRC patients with different expression of C3. (G) OS and RFS in CRC patients with different expression of SFTPA2. (H) OS and RFS in CRC patients with different expression of SFTPD.

We next assessed which of these candidate genes have prognostic importance. Kaplan–Meier survival curves (Figure 2) revealed that high expression of CCL18 was significantly associated with worse OS and shorter RFS in CRC patients (log‐rank *p* < 0.01). MMP3 showed a trend toward poor prognosis as well, though less pronounced. In contrast, high expression of surfactant genes SFTPA2/SFTPD (which were downregulated in metastases) correlated with better survival, consistent with their loss in aggressive disease. These data highlighted CCL18 as a strong candidate gene functionally relevant to CRC progression. Given its upregulation in metastases and prognostic value, we chose to focus on CCL18 in subsequent analyses.

### 3.3. Expression of Core Genes in CRC

**Figure 3:**
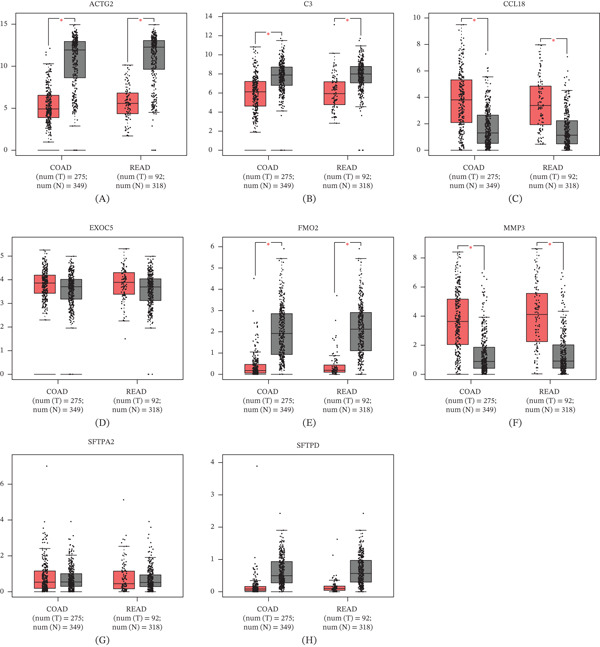
Expression of core genes in colorectal cancer. (A) The relative mRNA expression of ACTG2 in TCGA and relative normal tissues in GTEx. (B) The relative mRNA expression of C3 in TCGA and relative normal tissues in GTEx. (C) The relative mRNA expression of CCL18 in TCGA and relative normal tissues in GTEx. (D) The relative mRNA expression of EXOC5 in TCGA and relative normal tissues in GTEx. (E) The relative mRNA expression of FMO2 in TCGA and relative normal tissues in GTEx. (F) The relative mRNA expression of MMP3 in TCGA and relative normal tissues in GTEx. (G) The relative mRNA expression of SFTPA2 in TCGA and relative normal tissues in GTEx. (H) The relative mRNA expression of SFTPD in TCGA and relative normal tissues in GTEx.

To further explore the expression of core genes in CRC, we analyzed the expression profiles of CRC patients in TCGA and relative normal tissues in GTEx by GEPIA, and assessed the mRNA transcript levels of eight core genes. The results revealed that only CCL18 and MMP3 were specifically highly expressed in CRC tissues, whereas ACTG2, C3, and FMO2 showed a low expression profile; in addition, SFTPA2, SFTPD, and EXOC5 were not significantly differentially expressed in CRC tissues versus normal tissues. Jointly analyzing the prognosis of patients and on the expression difference, we selected CCL18 for the next analysis. (Figure 3).

### 3.4. Enrichment Analysis Links CCL18 to Immune and Metastatic Pathways

To hypothesize CCL18′s role, we performed single‐gene GSEA on TCGA CRC data stratified by high versus low CCL18 expression. Figure 4A illustrates that samples with high CCL18 had enrichment of gene sets related to immune suppression, invasion, and EMT. For example, NF‐kappa B signaling, inflammatory response, and cell adhesion molecules pathways were upregulated in the CCL18 high subset (FDR < 0.01), suggesting that high CCL18 expression is linked to a less immunoreactive microenvironment. These results align with known functions of CCL18 in promoting tumor invasion and dampening adaptive immunity.

Figure 4Functional enrichment analysis of CCL18 in the CRC. (A) Single‐gene GO and GSEA enrichment analyses of CCL18 through TCGA samples. (B) The immune infiltration score of CCL18 in the COAD and READ data of TCGA. (C) The immune cell infiltration score of CCL18 in the COAD and READ data of TCGA.

**Figure:**
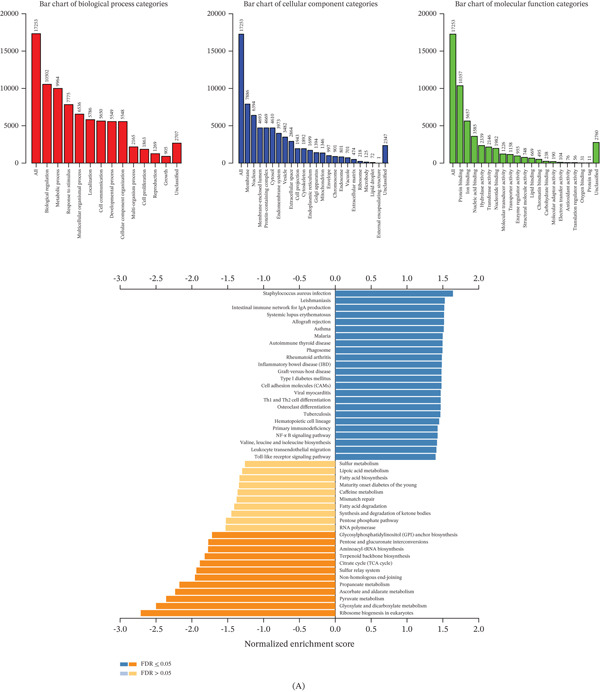
(A)

**Figure:**
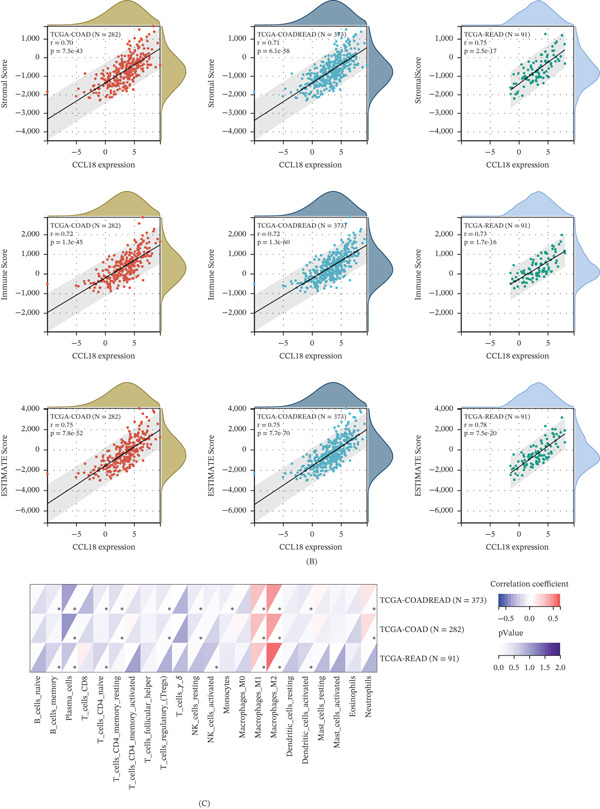
(B)

CCL18 is a chemokine that is mainly secreted by macrophages. Tumors do not exist in the body only as tumor cells, but have a complex TME. We speculate that CCL18 is derived from TAMs. We analyzed the immune infiltration score of CCL18 in the COAD and READ data of TCGA. We calculated Pearson′s correlation coefficients between genes and immune infiltration scores in individual tumors to identify significantly correlated immune infiltration scores, and ultimately, we observed that the expression of CCL18 was associated with immune infiltration in CRC. (Figure 4B) Further, we analyzed the immune cell infiltration score; we calculated the Pearson′s correlation coefficient between the CCL18 gene and immune cell infiltration score in CRC to determine the significant correlation of the immune infiltration score. Ultimately, we observed that CCL18 expression correlated most strongly with M2‐polarized macrophages (anti‐inflammatory and protumorigenic). (Figure 4C).

CCL18 stands out as a TAM‐associated chemokine upregulated in lung metastases and likely driving prometastatic programs. This provided a rationale for further biological experiments to determine if CCL18 actively promotes metastatic behavior in CRC cells.

### 3.5. Tumor Cells Lack Intrinsic CCL18 Expression

Before attributing functional effects to TAM‐derived CCL18, we confirmed that CRC tumor cells themselves do not produce meaningful CCL18. Analysis of the CCLE dataset (including CRC cell lines) showed nearly undetectable CCL18 mRNA in all epithelial cancer cell lines examined. This finding was corroborated by the Human Protein Atlas, which reports CCL18 protein is not expressed by carcinoma cells but is enriched in tumor‐infiltrating immune cells [17]. In our own lab, we performed RT‐PCR on several CRC cell lines (HCT116, SW480, and SW620) and consistently failed to amplify CCL18 transcripts. These results confirm that CCL18 is not an autocrine product of CRC epithelial cells but rather is exogenous to tumor cells, stemming from immune infiltrates. Therefore, any effects of CCL18 on CRC cells in our coculture experiments can be ascribed to macrophage‐derived CCL18, without confounding by tumor cell secretion.

### 3.6. M2‐Polarized Macrophages Secrete CCL18 and Enhance CRC Invasiveness

**Figure 5:**
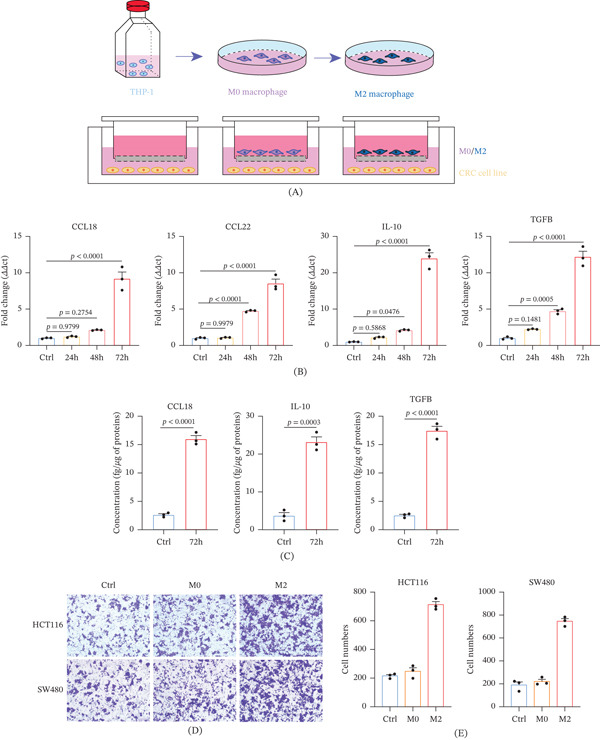
M2‐polarized macrophages promote CRC cell invasion. (A) The polarization process of THP‐1 and the establishment of a coculture system. (B) The relative mRNA expression of M2 polarization markers at different points in time. (C) The relative protein expression of M2 polarization markers at different points in time. (D) Transwell assay was used to analyze the effect of macrophages in different polarization states on the migration of CRC cells. (E) Statistical analysis of the effect of macrophages in different polarization states on the migration of CRC cells.

We conjectured that in CRC, CCL18 is mainly derived from M2‐polarized macrophages. We incubated THP‐1 macrophages in the M0 state with IL‐4 and IL‐13 at 20 ng/mL for 24, 48, or 72 h to test this conjecture. The M2 phenotype was characterized by examining the mRNA and protein abundance of several M2 markers. After 24 h, there was a slight increase in TGF‐*β* and IL‐10 expression, but not in CCL18 and CCL22. After 48 and 72 h, the mRNA abundance of all M2 markers increased (Figure 5B). ELISA confirmed the protein expression patterns of TGF‐*β*, IL‐10, and CCL18 (Figure 5C).

To see how M2 macrophages affect the ability of colon cancer cells to spread, we put M2 macrophages and colon cancer cells together in a special chamber. M2 cells were placed on membranes with 0.4‐*μ*m pores that allowed soluble factors to pass through but not cells. M2 and M0 macrophages were differentiated and polarized on the same day. Macrophages were cocultured with CRC cells for 24 h. At the end of the coculture, CRC cells in six‐well plates were collected, counted, relaid in chambers, and subjected to invasion assays, with the control group being CRC cells that were not cocultured. It was found that compared with normal CRC cells, CRC cells cocultured with M2‐polarized THP1 were more likely to pass through the chambers, whereas M0 did not change significantly (Figure 5D–E). This suggests that M2‐polarized macrophages can promote the invasive metastatic ability of CRC cells.

### 3.7. CCL18 Promotes CRC Cell Invasion and Proliferation in the Tumor Immune Microenvironment

**Figure 6:**
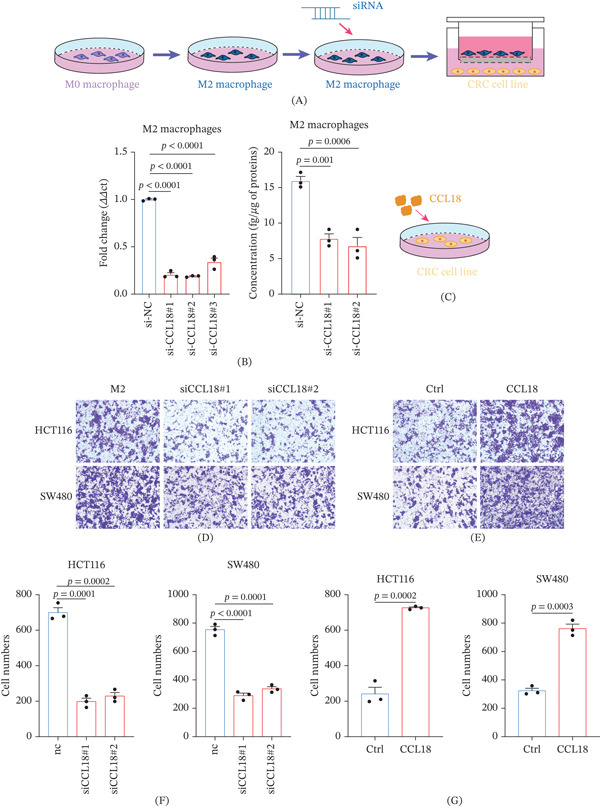
CCL18 promotes CRC cell invasion in the tumor immune microenvironment. (A) The THP‐1 was polarized and then subjected to knockdown treatment as well as the construction of a coculture system. (B) RT‐qPCR was utilized to evaluate the knockdown efficiency of CCL18 mRNA in M2 THP‐1 cells. (C) Treat CRC cells with recombinant CCL18 to remove interference from other factors. (D) Transwell assay was used to analyze the effect of CCL18 knockdown of M2 THP‐1 cells on the migration of CRC cells. (E) Transwell assay was used to analyze the effect of recombinant CCL18 treatment on the migration of CRC cells. (F) Statistical analysis of the effect of CCL18 knockdown of M2 THP‐1 cells on the migration of CRC cells. (G) Statistical analysis of the effect of recombinant CCL18 treatment on the migration of CRC cells.

To confirm that it is CCL18, but not other cytokines in the tumor immune microenvironment, that significantly enhances tumor invasive metastasis, we interfered with CCL18 expression in M2‐polarized macrophages (Figure 6A,C). Knockdown of CCL18 showed a trend toward reduced mRNA and protein expression (Figure 6B). M2‐polarized macrophages with knocked‐down CCL18 were cocultured with CRC cells, and the invasive metastatic ability of CRC cells was subsequently examined using a transwell assay, and it was found that the invasive and metastatic abilities of CRC cells were inhibited (Figure 6D,F). On the other hand, CRC cells were treated with recombinant CCL18, and the invasive metastatic ability of CRC cells was also detected using a transwell assay, which revealed that their invasive and metastatic abilities were enhanced (Figure 6E,G).

**Figure 7:**
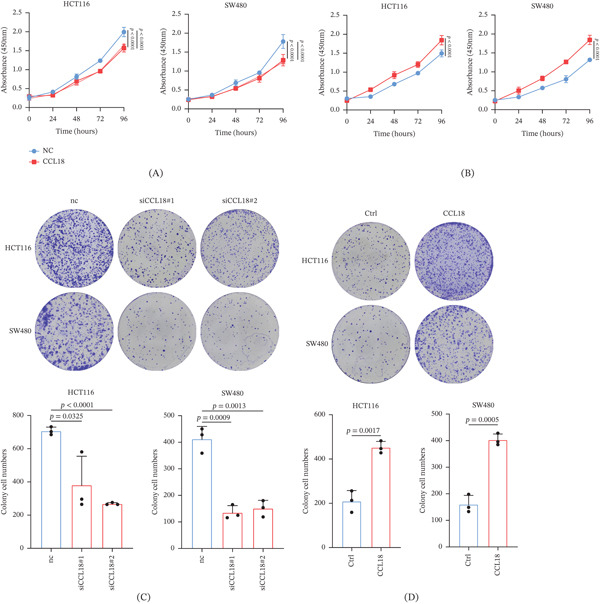
CCL18 promotes CRC cell proliferation in the tumor immune microenvironment. (A, B) CCK8 assay was used to analyze the effect of CCL18 knockdown of M2 THP‐1 cells on the proliferation of CRC cells. (C, D) Colony formation assays were used to analyze the effect of CCL18 knockdown of M2 THP‐1 cells or recombinant CCL18 treatment on the proliferation of CRC cells.

While metastasis is the primary focus, we recognize that any factor promoting metastasis could also influence primary tumor growth or postsurgical recurrence. To address this question, we examined CRC‐cell proliferation under the conditions described above in the presence of CCL18. Preliminary data show that exposure to CCL18 modestly increases the proliferation rate of CRC cells, whereas proliferation is suppressed when the cells are cultured within an immune microenvironment containing M2 macrophages in which CCL18 has been silenced (Figure 7). These data suggest that, beyond facilitating invasion, CCL18 may confer a growth advantage to tumor cells, potentially aiding micrometastatic colony expansion and tumor relapse.

**Figure 8:**
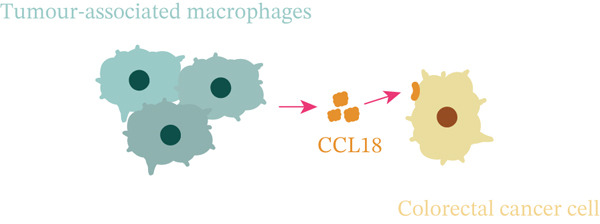
CCL18 in the tumor microenvironment enhanced the invasive metastatic ability of the tumor.

In summary, our results thus far confirm that M2 TAM‐derived CCL18 is a key promoter of CRC cell invasive metastasis in the lung microenvironment. Figure 8 presents a working model: CRC cells in the lung metastatic niche encounter CCL18 secreted by infiltrating M2 macrophages, which in turn activates signaling pathways in cancer cells (and possibly stromal cells) that drive EMT, invasion, survival, and proliferation. These findings form the basis for an expanded discussion on CCL18′s mechanism of action and clinical implications.

## 4. Discussion

In this study, we identified CCL18 as a pivotal gene associated with CRC lung metastasis and demonstrated that TAM‐derived CCL18 can potently augment the invasive and proliferative behavior of CRC cells. Our integrated approach highlights a critical crosstalk between TAMs and cancer cells in the metastatic microenvironment.

Our work reinforces the concept that TME plays a decisive role in metastasis. Tumors are infiltrated by immune cells that can either attack the cancer or inadvertently aid its progression. Macrophages are especially influential; they constitute a major component of the tumor stroma and exhibit functional plasticity ranging from proinflammatory (M1‐like) to anti‐inflammatory (M2‐like) states. Consistent with previous reports, we found that CRC lung metastases are enriched in M2‐polarized TAMs, which correlated with high CCL18 expression and poor patient outcomes. M2 TAMs secrete a milieu of cytokines, growth factors, and chemokines that condition the TME to favor tumor survival and immune evasion. CCL18 is a prime example of such an M2‐associated factor: It not only serves as a marker of M2 TAM infiltration but also actively contributes to the immunosuppressive, protumor environment.

One of the hallmark functions of CCL18 is its ability to induce immune tolerance and escape. CCL18 drives Treg differentiation and recruitment in tumors, and fosters a feed‐forward loop polarizing macrophages toward a tumor‐promoting M2 phenotype. In other words, high CCL18 can skew the local immune balance away from antitumor effector cells and toward immunosuppressive actors, thereby blunting the immune system′s ability to eliminate cancer cells. Our finding of positive correlations between CCL18 and Treg/MDSC signatures in CRC tumors aligns with this immunosuppressive role [18, 25]. Moreover, CCL18 was recently implicated in immune checkpoint regulation. Clinically, high serum or tissue CCL18 might predict poor response to immune checkpoint inhibitors due to the abundance of TAMs and Tregs it signifies [26–28].

Beyond immune modulation, CCL18 exerts direct effects on tumor biology that facilitate metastasis [29, 30]. CCL18 from TAMs promotes breast cancer metastasis by enhancing cancer cell invasiveness. Mechanistically, CCL18 binds to a receptor on cancer cells and triggers intracellular signaling that leads to integrin *β*1 clustering on the cell surface. This increases cancer cells′ adhesiveness to the extracellular matrix (ECM), thereby promoting migration and transendothelial invasion. We observed a parallel in CRC: CCL18 exposure increased CRC cell invasion through Matrigel, consistent with improved ECM attachment and motility. It is plausible that CRC cells also express a functional CCL18 receptor. It is noteworthy that CCR8 is expressed on certain tumor cells and Tregs, and is being explored as an immunotherapy target. Whether CCR8 or PITPNM3 mediates CCL18′s effects in CRC requires further study; possibly both receptors could be involved in different contexts.

Interestingly, not all evidence points uniformly in one direction; the tumor context can modulate CCL18′s impact. Nonetheless, the overwhelming theme in solid tumors is that CCL18 signifies a protumoral TAM presence and contributes to metastasis and immune evasion. Our CRC lung metastasis study adds to this body of evidence, underscoring that CCL18′s role is conserved across cancer types, yet it also emphasizes the need to consider tissue‐specific nuances [31, 32].

CRC provides a unique scenario to examine CCL18 because metastatic spread can occur to multiple organs with differing microenvironments. Liver metastases of CRC have been extensively studied, and TAMs are known contributors to liver metastatic niches via CCL2/CCR2 and other factors. Lung metastases, however, have distinct challenges such as higher immune surveillance (lungs are rich in immune cells filtering blood) and a different stromal composition. Our study specifically highlights CCL18 in the context of CRC lung metastasis, which has not been thoroughly explored before. We demonstrate that CCL18 is highly upregulated in CRC lung metastatic lesions relative to primary tumors, indicating that the lung TME strongly induces or selects for CCL18‐expressing TAMs. This could be due to lung‐specific signals. Additionally, we showed that CRC lung metastases with high CCL18 had particularly aggressive features. This suggests that CCL18 might be a key determinant of why some CRC metastases flourish in the lung. It could explain, in part, the clinical observation that patients with robust immune reactions sometimes control pulmonary metastases better.

From a clinical perspective, CCL18 could serve as a biomarker for CRC patients at risk of lung metastasis. For instance, one could envision testing resected primary CRC tumors for CCL18+ TAM density or CCL18 gene expression; a high level might indicate a “premetastatic niche” already forming in the lungs, thus alerting clinicians to monitor the lungs closely for metastases. In fact, our identification of CCL18 aligns with another recent bioinformatics study that found CCL18 as a hub gene in CRC lung metastasis and experimentally confirmed that one of them, SFTPD, can facilitate metastasis. Those authors proposed CCL18 as a lung‐tropism–associated gene, which our work now validates functionally.

Moreover, targeting CCL18 or its downstream pathways could be therapeutically beneficial in CRC with lung metastases. Since CCL18 is not produced by the cancer cells, therapies would be aimed at the stromal compartment, for example, depleting TAMs, repolarizing TAMs from M2 to M1, or neutralizing CCL18 itself. Although broad TAM depletion can be harmful, strategies to selectively reprogram TAMs are in development. If we can convert an M2‐dominated TME to an M1‐dominated one, CCL18 levels would drop and the immune system could regain control. Along these lines, CCR8 inhibitors/antibodies are being investigated in early trials and might enhance antitumor immunity in combination with checkpoint blockade. Alternatively, small molecule inhibitors or monoclonal antibodies against PITPNM3 could be explored to prevent CCL18 from activating cancer cells. The precedent in breast cancer, where a CCL18 antagonist suppressed metastasis, is encouraging. In CRC, no such antagonist has been tested yet, but our results would support such an approach.

We also acknowledge that CCL18 might interplay with other CRC metastasis factors. For example, MMP3 was another gene coupregulated with CCL18 in lung metastases. It is conceivable that TAMs cosecrete MMPs along with CCL18, and together they facilitate metastatic colonization. These cofactors might work in concert under the orchestration of TAMs. Future work dissecting the CCL18 signaling network in CRC cells will clarify how it intersects with pathways like NF‐*κ*B, STAT3, or others commonly activated in metastasis.

Although our study provides a comprehensive analysis, there are some limitations to note. First, our in vivo validation is pending. We plan to perform mouse models of CRC metastasis to confirm that knocking out or inhibiting CCL18 can reduce lung metastatic burden. In the current work, we relied on human clinical data and in vitro assays. Second, the precise receptor and signaling pathway for CCL18 in CRC cells remain to be elucidated. We infer from other cancers that PITPNM3 or CCR8 might be involved; confirming this in CRC cells will be important, as it dictates what drug strategy could work. Third, our coculture experiments used a THP‐1 cell line model for TAMs. Although convenient and reproducible, primary macrophages from CRC patients could provide additional insights, as they might have different polarization dynamics. We did show concordance between THP‐1–derived M2 and patient TAM behavior, but patient‐derived macrophages in a 3D ex vivo lung tissue model would strengthen translational relevance.

Given its multifaceted protumor roles, such as immune suppression, EMT induction, invasion, angiogenesis, and possibly metabolic reprogramming, CCL18 represents an attractive therapeutic target or biomarker [33, 34]. Encouragingly, CCL18 is secreted and mostly extracellular, making it accessible to antibodies or small molecules. Furthermore, patients could be monitored for therapy response by measuring serum CCL18 levels [35, 36]. If an anti‐CCL18 therapy is given, drops in serum CCL18 might correlate with TAM reprogramming and tumor regression.

It is also feasible to combine CCL18‐targeted therapy with immunotherapy. By neutralizing CCL18, one might reduce Treg recruitment and M2 polarization, thereby flipping the immune microenvironment to a state more amenable to T‐cell mediated killing. This could synergize with checkpoint inhibitors in CRC. Diminishing CCL18 could be part of a strategy to turn a “cold” tumor “hot” by removing the TAM/Treg brakes on the immune system.

In conclusion, our discussion emphasizes that CCL18 is a central player in tumor progression across multiple cancers, and our data establish its significance in CRC lung metastasis. We have shown that focusing on the M2 TAM–CCL18 axis yields insights into how CRC cells metastasize to the lungs. This axis is a potential vulnerability of the tumor: Therapies disrupting TAM recruitment, TAM polarization, or CCL18 signaling itself could all impair the metastatic cascade. The next steps should include validating these therapeutic opportunities in preclinical models and perhaps retrospective clinical analyses to confirm that patients with high CCL18 indeed fare worse, which would justify stratifying patients for TAM‐targeted treatments.

## 5. Conclusion

In summary, our study identifies CCL18 as a key facilitator of CRC lung metastasis. Through integrated bioinformatics and experimental validation, we demonstrate that CCL18 is markedly upregulated in CRC lung metastatic tissues and is associated with poor patient prognosis. CCL18 shapes a prometastatic TME by promoting immune evasion and directly enhancing cancer cell invasive and EMT capabilities. Coculture experiments confirmed that macrophage‐derived CCL18 significantly increases CRC cell invasion and proliferation in vitro. These findings suggest that CCL18 drives tumor progression and metastatic outgrowth in the lungs, making it a promising candidate for therapeutic intervention.

From a clinical standpoint, CCL18 could serve as a prognostic biomarker and a potential drug target in metastatic CRC. Patients with CCL18‐rich tumors might benefit from therapies targeting the TAM/CCL18 axis. What is particularly novel here is elucidating CCL18′s role in CRC’s pulmonary metastases, a context previously under‐investigated. By highlighting the importance of the M2 TAM–CCL18–cancer cell interplay, we pave the way for new strategies to disrupt this crosstalk.

In conclusion, our study provides a comprehensive insight into how the tumor immune microenvironment epitomized by M2 macrophage‐derived CCL18 fuels CRC metastasis. These insights reinforce the paradigm that effective cancer therapy must go beyond cancer cells alone and also target the supportive ecosystem enabling their spread. CCL18 represents a convergence point of protumor inflammation, immune suppression, and metastatic activation. Therapeutic efforts to neutralize CCL18 or its effects could significantly impair metastatic progression and improve outcomes for CRC patients, especially those suffering from lung metastases. Our findings not only advance the fundamental understanding of CRC metastasis but also have tangible implications for developing immune microenvironment‐focused interventions in the fight against metastatic cancer.

## Author Contributions

Qingcheng Xia, Shuting Yin, and Jiping Chen contributed equally to this work.

## Funding

This study was supported by Jiangsu Provincial Health Commission Scientific Research Project (H2023058).

## Conflicts of Interest

The authors declare no conflicts of interest.

## Data Availability

The data that support the findings of this study are available from the corresponding author upon reasonable request.
